# The effect of auto-verification on turnaround time for clinical chemistry and immunoassay tests

**DOI:** 10.5937/jomb0-61380

**Published:** 2026-06-16

**Authors:** Havva Yasemin Çinpolat

**Affiliations:** 1 Çanakkale Onsekiz Mart University, Faculty of Medicine, Department of Medical Biochemistry, Çanakkale, Türkiye

**Keywords:** algorithms, autoverification, information technology, quality improvement, turnaround time (TAT), algoritmi, auto-verifikacija, informaciona tehnologija, unapređenje kvaliteta, vreme izdavanja rezultata (TAT)

## Abstract

**Background:**

Auto-verification is increasingly recognised as a key tool for improving quality and efficiency in clinical laboratories. This study aimed to investigate the impact of an auto-verification system implemented for clinical chemistry and immunoassay tests on turnaround time (TAT) in a tertiary-care medical biochemistry laboratory.

**Methods:**

This study was conducted in the Medical Biochemistry Laboratory of Çanakkale Onsekiz Mart University Hospital, a tertiary healthcare institution. Clinical chemistry and immunoassay tests were automated and verified using the navify® Lab Operations middleware (Roche Diagnostics, Germany), integrated with the MIA-MED Laboratory Information System (MIA Technology, Türkiye). Algorithms were developed in accordance with CLSI AUTO10-A and AUTO 15 guidelines, incorporating rules for quality control, serum indices, analyser flags, delta checks, critical values, consistency checks, and analytical measurement intervals, as well as recommendations from the national health authorities. Validation of the algorithms was carried out using both simulated and patient data. The proportion of results exceeding predefined TAT targets was compared before and after auto-verification implementation using the chi-square test. p&lt; 0.05 was considered statistically significant.

**Results:**

Overall, 71% of test results and 21% of tube-based results were verified automatically. Median TAT was reduced by 6 minutes for emergency tests and 12 minutes for routine tests. The proportion of results exceeding the TAT threshold decreased significantly from 6.4% before auto-verification to 5.8% after auto-verification implementation (p&lt; 0.001).

**Conclusions:**

Auto-verification, with clearly defined and validated rules, enhances both the reliability and timeliness of laboratory results, thereby supporting quality improvement initiatives in clinical laboratories.

## Abstract (sr)

**Uvod:**

Auto-verifikacija se sve vise prepoznaje kao ključni alat za unapređenje kvaliteta i efikasnosti u kliničkim laboratorijama. Cilj ove studije bio je da ispita uticaj sistema auto-verifikacije, primenjenog za testove kliničke hemije i imunoodbrambene (imunoesej) analize, na vreme izdavanja rezultata (TAT) u tercijarnoj zdravstvenoj ustanovi iz oblasti medicinske biohemije.

**Metode:**

Studija je sprovedena u Laboratoriji za medicinsku biohemiju Bolnice Çanakkale Onsekiz Mart University, tercijarne zdravstvene ustanove. Testovi kliničke hemije i imunoeseja bili su podvrgnuti automatskoj verifikaciji korišćenjem navify® Lab Operations middleware sistema (Roche Diagnostics, Nemačka), integrisanog sa laboratorijskim informacionim sistemom MIA-MED (MIA Technology, Turska). Algoritmi su razvijeni u skladu sa CLSI AUTO10-A i AUTO 15 smernicama i uključivali su pravila za kontrolu kvaliteta, serumske indekse, oznake analizatora, delta provere, kritične vrednosti, provere konzistentnosti i opsege mernih intervala, kao i preporuke nacionalnih zdravstvenih vlasti. Validacija algoritama je sprovedena korisćenjem simuliranih i realnih podataka pacijenata. Procenat rezultata koji su prekoračili unapred definisane TAT ciljne vrednosti upoređen je pre i nakon uvođenja auto-verifikacije koristeći Hi-kvadrat test. Vrednost p&lt; 0,05 smatrana je statistički značajnom.

**Rezultati:**

Ukupno je 71% rezultata testova i 21% rezultata na nivou epruveta verifikovano automatski. Srednj'e vreme izdavanja rezultata smanjeno je za 6 minuta kod urgentnih i za 12 minuta kod rutinskih testova. Procenat rezultata koji su prekoračili TAT prag značajno je opao sa 6,4% pre auto-verifikacije na 5,8% nakon implementacije (p&lt; 0,001).

**Zaključak:**

Auto-verifikacija sa jasno definisanim i validiranim pravilima unapređuje pouzdanost i pravovremenost laboratorijskih rezultata, čime podržava inicijative za poboljšanje kvaliteta u kliničkim laboratorijama.

## Introduction

Patient test reports from clinical laboratories are widely used for diagnosis, hospitalisation, discharge, treatment decisions, and monitoring clinical status. As the demand for healthcare services has increased in recent years, the number and variety of tests that clinical laboratories must analyse have also grown accordingly. Manual verification of accurate and precise patient test results within an acceptable turnaround time (TAT) constitutes a critical part of the workload of clinical biochemistry specialists [1].

Before test results are sent to clinicians, they must be verified to detect any errors that may have occurred throughout the total testing process. This verification relies on human-centred mental algorithms. Evaluating multiple variables - such as the patient's age, clinical condition, diagnosis, sample collection time, sample receipt time in the laboratory, analysis-related warnings, panic values, and delta checks - renders the process subjective and heavily reliant on experience. Given the shortage of clinical biochemistry specialists and technicians in many laboratories, managing this entire process presents a significant challenge [2].

Auto-verification is a tool used in medium and large clinical laboratories that improves the overall testing process, particularly the post-analytical phase. It helps reduce the laboratory's workload and shortens TAT [3]. Middleware software facilitates this process by communicating between systems: it receives patient and sample data from the laboratory information system (LIS) and gathers test results along with analytical process data from laboratory instruments [4]. A specific set of rules, established by the laboratory and designed as algorithms, enables the software to release test results within a standardised procedure without human intervention [5].

The AUTO10-A and AUTO15 documents published by the Clinical Laboratory Standards Institute (CLSI) provide recommendations for rule definition, algorithm development, implementation, validation, and essential requirements [6][7]. These documents emphasise that each laboratory should tailor auto-verification processes to the specific needs of its patient population. Algorithms can be logically constructed to simulate human reasoning and should only permit auto-verification after acceptable quality control (QC) results have been confirmed. Patient-based QC can also be integrated into the workflow. Rules may incorporate serum indices, instrument or sample-related alerts, and patient data from the LIS - such as demographic information, diagnosis, and inpatient/outpatient status. Criteria within the algorithm may include: the result must fall within the instrument's analytical measurement range, not exceed defined critical values, and be proportionally consistent with related test results [8].

Another key consideration is defining the autoverification interval. There are various approaches to this. One method is using reference intervals or clinical decision levels to automatically release test results to the LIS without manual review [9]. Another involves using reference ranges combined with total allowable error (TEa) limits [10]. Laboratories may also establish auto-verification thresholds specific to their patient population by calculating the 2nd and 98th percentile intervals [11].

In 2018, the Department of Examination and Diagnostic Services of the Ministry of Health published recommendations on the use of auto-verification in clinical laboratories as part of the Rational Laboratory Project [12]. Although auto-verification has become more widespread in Turkey following these guidelines, there are still limited studies on its implementation. This study aimed to evaluate the outcomes of auto-verification, implemented for the first time in a university hospital, and to assess its impact on TAT before and after its adoption.

## Materials and methods

The Ethics Committee of Çanakkale Onsekiz Mart University approved the study with decision number 11/51 on 09/2023. Çanakkale Onsekiz Mart University Hospital, a tertiary healthcare institution with 535 beds, provides care for approximately 3,000 inpatients and 47,000 outpatients per month. The Clinical Biochemistry Laboratory processes around 270,000 tests monthly, primarily consisting of clinical chemistry and immunoassay tests.

Clinical chemistry tests were performed using cobas c702 and c501 autoanalyzers (Roche Diagnostics, Germany), while immunoassay tests were analysed using cobas e601 and e602 analysers (Roche Diagnostics, Germany). Two cobas 6000 and one cobas 8000 integrated and modular systems were utilised for both clinical chemistry and immunoassay testing. One of the cobas 6000 systems was dedicated primarily to emergency and intensive care unit services.

Auto-verification was carried out using the navify® Lab Operations middleware (Roche Diagnostics, Germany), integrated with the MIA-MED Laboratory Information System (MIA Technology, Türkiye).

### Measurands

The clinical chemistry tests included the following parameters: albumin, alkaline phosphatase (ALP), alanine aminotransferase (ALT), ammonia, amylase, anti-streptolysin O (ASO), aspartate aminotransferase (AST), calcium, chloride, cholesterol, cholinesterase, creatine kinase, creatinine, C-reactive protein (CRP), direct and total bilirubin, gamma-glutamyltransferase (GGT), glucose, high-density lipoprotein (HDL), iron, lactate, lactate dehydrogenase (LDH), low-density lipoprotein (LDL), lipase, magnesium, phosphate, potassium, rheumatoid factor (RF), sodium, total protein, triglyceride, unsaturated iron-binding capacity, urea, and uric acid.

The immunoassay tests included for auto-verification were: 25-hydroxy vitamin D3, alpha-fetoprotein, thyroglobulin antibody, thyroid peroxidase antibody, CA 125, CA 15-3, CA 19-9, carcinoembryonic antigen, CK-MB, cortisol, estradiol, ferritin, folate, free and total prostate-specific antigen (PSA), free triiodothyronine (fT3), free thyroxine (fT4), follicle-stimulating hormone (FSH), luteinising hormone (LH), pro-brain natriuretic peptide (pro-BNP), procalcitonin, progesterone, prolactin, parathyroid hormone (PTH), testosterone, thyroid-stimulating hormone (TSH), and vitamin B12.

### Algorithms

Auto-verification algorithms were developed using a set of rules that included QC and calibration warnings, serum indices, analyser flags/warnings, auto-verification limits, delta checks, critical values, consistency checks, and analytical measurement intervals, in accordance with CLSI AUTO10-A and CLSI AUTO15 guidelines [6][7]. Additionally, the recommendations issued by the Department of Examination and Diagnostic Services of the Ministry of Health regarding auto-verification in clinical laboratories were considered [12].

**Figure 1:**
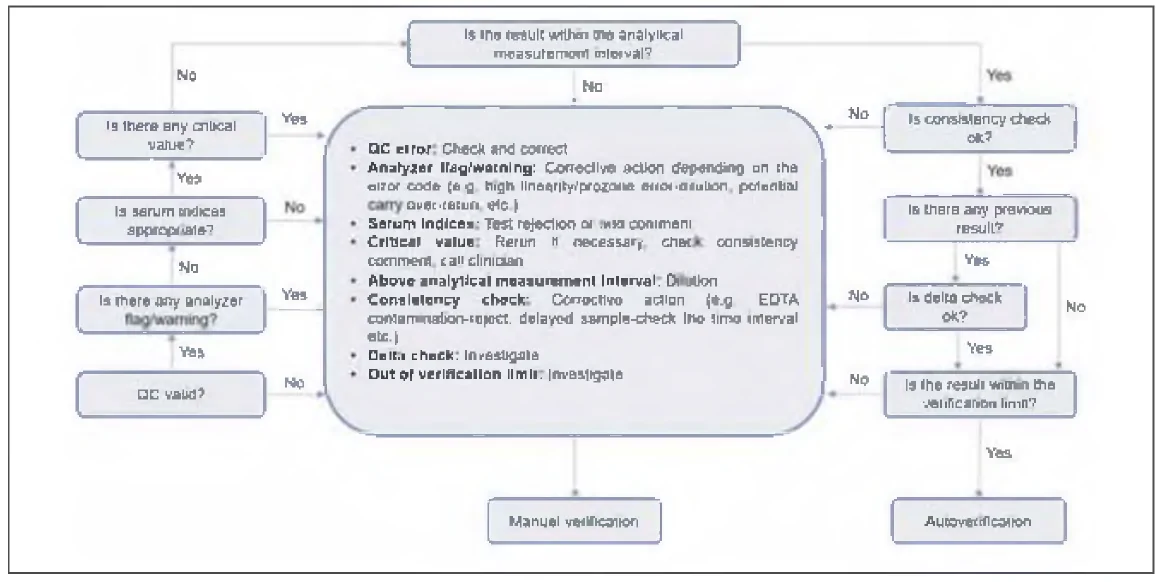
The flowchart of autoverification rules of the clinical chemistry and immunassay tests.

The main algorithm, generally applied across tests, is illustrated in Figure 1. All rules operated concurrently, and if any rule defined for a particular test was violated, the test result was forwarded to the clinical biochemistry specialist for manual verification, along with an indication of the rule that prevented auto-verification. Test-specific rules are summarised in Table 1 and Table 2.

**Table 1:** The rules for clinical chemistry tests. ALP alkaline phosphatase; ALT, alanine aminotransferase; ASO, anti streptolysin O; AST, aspartate aminotransferase; CK, creatine kinase; CRP C reactive protein; GGT, gamma-glutamyltransferase; HDL, high density lipoprotein; LDH; lactate dehydrogenase; LDL, low density lipoprotein; RF, rheumatoid factor; UIBC; unsaturated iron binding capasity; HL, high linearity; LL, low linearity; RI, reference interval; TEa, allowable total error; CDL, clinical decision limit

Test	Quality<br>control error	Analyser<br>flag	Serum<br>indices	Critical<br>value	Analytical measurement<br>interval	Consistency<br>check	Delta<br>check	Auto verification<br>limit
Albumin	+	+	+	-	HL, LL	+	13.2%	RI ± TEa
ALP	+	+	+	-	HL, LL	+	20.2%	RI ± TEa
ALT	+	+	+	-	HL, LL	+	54.3%	RI ± TEa
Ammonia	+	+	+	+	HL	-	-	RI ± TEa
Amylase	+	+	+	+	HL, LL	-	25.1%	RI ± TEa
ASO	+	+	+	-	HL	-	-	RI
AST	+	+	+	-	HL, LL	+	35.0%	RI ± TEa
Calcium	+	+	+	+	HL, LL	+	8.7%	RI ± TEa
Chloride	+	+	+	+	HL, LL	-	5.0%	RI
Cholesterol	+	+	+	-	HL, LL	+	19.0%	CDL ± TEa
Cholinesterase	+	+	+	-	HL, LL	-	-	RI ± TEa
CK	+	+	+	+	HL, LL	-	63.6%	RI ± TEa
Creatinine	+	+	+	+	HL, LL	-	17.9%	RI ± TEa
CRP	+	+	+	-	HL	-	95.1%	RI ± TEa
Direct bilirubin	+	+	+	+	HL	+	102.5%	RI ± TEa
GGT	+	+	+	-	HL, LL	-	38.2%	RI ± TEa
Glucose	+	+	+	+	HL, LL	+	16.3%	CDL ± TEa
HDL	+	+	+	-	HL, LL	+	21.1%	CDL ± TEa
Iron	+	+	+	-	HL, LL	-	74.1%	RI ± TEa
Lactate	+	+	+	-	HL, LL	-	-	RI ± TEa
LDH	+	+	+	-	HL, LL	-	26.1%	RI ± TEa
LDL	+	+	+	-	HL, LL	+	23.6%	CDL ± TEa
Lipase	+	+	+	-	HL, LL	-	26.9%	RI ± TEa
Magnesium	+	+	+	+	HL, LL	+	11.8%	RI ± TEa
Phosphate	+	+	+	+	HL, LL	-	24.2%	RI ± TEa
Potassium	+	+	+	+	HL, LL	+	14.4%	RI
RF	+	+	+	-	HL	-	-	RI ± TEa
Sodium	+	+	+	+	HL, LL	-	5.0%	RI
Total bilirubin	+	+	+	+	HL	+	61.6%	RI ± TEa
Total protein	+	+	+	-	HL, LL	+	9.9%	RI ± TEa
Triglyceride	+	+	+	-	HL, LL	+	55.6%	CDL ± TEa
UIBC	+	+	+	-	HL, LL	-	-	RI ± TEa
Urea	+	+	+	-	HL, LL	-	34.6%	RI ± TEa
Uric acid	+	+	+	-	HL, LL	-	24.8%	RI ± TEa

**Table 2:** The rules for immunassay tests. AFP alphafetoprotein; anti-Tg, thyroglobulin antibody; anti-TPO, thyroid peroxidase antibody; CA, cancer antigen; CEA, carcinoem-bryonic antigen; CK, creatine kinase; PSA, prostate specific antigen; T3, tri-iodothyronine; T4, thyroxine; FSH, follicle stimulating hormone; LH, luteinising hormone; BNP brain natriuretic peptide; PTH, parathyroid hormon; TSH, thyroid stimulating hormone; HL, high limit; LL, low limit; RI, reference interval; TEa, allowable total error; CDL, clinical decision limit

Test	Quality<br>control<br>error	Analyser<br>flag	Serum<br>indices	Critical<br>value	Analytical<br>measurement<br>interval	Consistency<br>check	Delta<br>check	Auto verification<br>limit
25-OH<br>vitamin D3	+	+	-	-	HL, LL	-	-	CDL ± TEa
AFP	+	+	-	-	HL, LL	-	15.5%	RI ± TEa
Anti-Tg	+	+	-	-	HL, LL	-	-	RI
Anti-TPO	+	+	-	-	HL, LL	-	-	RI
CA 125	+	+	-	-	HL, LL	-	25.6%	RI ± TEa
CA 15-3	+	+	-	-	HL, LL	-	22.9%	RI ± TEa
CA 19-9	+	+	-	-	HL, LL	-	14.0%	RI ± TEa
CEA	+	+	-	-	HL, LL	-	20.5%	RI ± TEa
CK-MB	+	+	-	-	HL, LL	-	-	RI ± TEa
Cortisol	+	+	-	-	HL, LL	-	45.6%	RI ± TEa
Estradiol	+	+	-	-	HL, LL	-	42.8%	RI ± TEa
Ferritin	+	+	-	-	HL, LL	-	36.7%	RI ± TEa
Folat	+	+	-	-	HL, LL	-	69.3%	RI ± TEa
Free PSA	+	+	-	-	HL, LL	-	23.5%	RI ± TEa
Free T3	+	+	-	-	HL, LL	-	15.6%	RI ± TEa
Free T4	+	+	-	+	HL, LL	+	16.5%	RI ± TEa
FSH	+	+	-	-	HL, LL	-	34.8%	RI ± TEa
LH	+	+	-	-	HL, LL	-	63.3%	RI ± TEa
Pro BNP	+	+	-	-	HL, LL	-	-	RI
Procalcitonin	+	+	-	-	HL, LL	-	-	CDL
Progesteron	+	+	-	-	HL, LL	-	51.9%	RI ± TEa
Prolactin	+	+	-	-	HL, LL	-	81.9%	RI ± TEa
PTH	+	+	-	-	HL, LL	-	44.0%	RI ± TEa
Testosteron	+	+	-	-	HL, LL	-	37.0%	RI ± TEa
Total PSA	+	+	-	-	HL, LL	-	19.8%	RI ± TEa
TSH	+	+	-	-	HL, LL	+	49.5%	RI ± TEa
Vitamin B12	+	+	-	-	HL, LL	-	44.3%	RI ± TEa

QC Error: Internal quality control (QC) data routinely applied in the laboratory were transferred from the analyser to the middleware and evaluated using Westgard multi-rules with Levey-Jennings charts. If any rule was violated, auto-verification was halted.

Analyser Flag/Warning: Error codes or warnings - such as prozone effect, sample clotting, or carryover - generated by the analyser were transferred to the middleware. These typically indicated mechanical malfunctions involving the reagents, sample, or analyser, or the presence of factors interfering with reaction formation or termination. Upon detection, autoverification was stopped.

Serum indices: Parameter-specific serum index thresholds, as specified in the kit inserts, were defined for clinical chemistry tests. If an index value exceeded the defined limit or if the serum index could not be determined, the analyser generated an error code, and the affected parameter was referred for manual verification.

Critical values: Critical values were defined based on the recommendations of the Department of Examination and Diagnostic Services of the Ministry of Health, in conjunction with input from clinicians [13]. When a critical value was detected, a flag was generated, and the result was manually verified after the clinician was notified.

Analytical measurement interval: The results exceeding linearity were diluted automatically or manually.

Consistency check: Consistency between at least two related test results from the same patient is assessed, and auto-verification is halted if a discrepancy is detected. Discordant results are flagged under the following conditions [8]:

- Albumin/Total protein ratio is >1 or <0.25
- AST/ALT ratio is >1 or <0.25
- HDL + LDL + (Triglyceride/5) > Total cholesterol
- HDL/Total cholesterol > 0.75
- Direct bilirubin > Total bilirubin
- Unusual hypothalamic-pituitary-thyroid axis patterns, such as both TSH and fT4 above the upper reference limit, or both below the lower reference limit

EDTA contamination alert: Potassium >7 mmol/L accompanied by any of the following - Calcium <8 mg/dL, ALP <50 U/L, or Magnesium <1.2 mg/dL

Delayed sample alert: Glucose <40 mg/dL, Potassium >6 mmol/L, and Hemolysis Index <50, when occurring together.

In any of these cases, the results are flagged and sent for manual verification.

Delta Check: The Reference Change Value (RCV), which incorporates both analytical variation (CVa) and within-subject biological variation (CVw), was used to evaluate the significance of changes between current and previous test results. CVa was calculated from the internal quality control data collected over the previous three months. Because a result could originate from any of several analysers, a pooled CVa was used. CVw values were obtained from the European Federation of Clinical Chemistry and Laboratory Medicine (EFLM) and Westgard biological variation databases [14][15][16].

A bidirectional Z-score corresponding to a 95% confidence interval was applied in the calculation of RCV, using the following formula:

RCV=Z^*2^{1/2_*}(CV_A^{2}+CV_W^{2})^{1/2}

If a previous result within the past 90 days is available and the percentage change between the current and previous results exceeds the RCV calculated for the respective parameter, the middleware generates a flag and forwards the result for manual verification.

Auto-Verification Limits: The decision limits for auto-verification were primarily established by extending the lower and upper reference intervals using the TEa for most routine clinical chemistry analytes that had well-established biological variation data and broadly accepted TEa limits. For analytes with clearly defined clinical decision limits that directly guide treatment decisions (e.g., glucose, lipids), the verification limits were constructed by expanding the clinical decision levels using TEa. For tests without universally accepted TEa values, only reference intervals or clinical decision limits were used to define the autoverification limits.

TEa values were primarily derived from CLIA proficiency testing criteria and analytical performance specifications based on biological variation [14][15][16][17]. Test results were automatically verified if they fell within the defined limits and no other rule violations were present.

Notable Exceptions: If a primary test meets all criteria and is auto-verified, any associated calculated test is also auto-verified without being submitted for manual review. For parameters such as CRP where low values are expected in healthy individuals, results below the lower limit of the measurement interval are automatically verified - provided no other rule violations are present. Additionally, if a test result is not verified at the time of its initial reporting, any subsequent repetition of the test is also directed to manual verification, regardless of the outcome.

### Validation of the algorithm

The algorithms were validated in accordance with CLSI AUTO10-A guidelines [6]. The validation process was based on both simulated and actual patient results. Initially, simulated datasets were created to represent rule-based conditions - particularly at decision thresholds and both within and outside those thresholds - for all relevant tests. These simulations were used to confirm that the algorithms functioned as intended.

Validation also included real patient results, encompassing values inside and outside both the reference and auto-verification limits. The algorithms were tested using challenging patient specimens with characteristics such as haemolysis, and those containing very low or very high analyte concentrations. In cases where suitable patient specimens were not available, spiked or manipulated samples - either containing interfering substances or externally added analytes - were prepared to simulate extreme test conditions.

As part of the validation process, the middleware system began transferring information to the LIS, indicating whether a result could be auto-verified and, if so, the reason for any rule violation, which appeared as a comment in a separate column.

A comprehensive checklist was created, and validation was conducted on a test-by-test basis lasting about six months. Auto-verification in the live system was temporarily withheld for tests involving consistency checks until all related parameters had been successfully validated. During the validation period, almost 15,000 patient reports corresponding to approximately 1,300,000 individual test results were evaluated in the live system. Auto-verification for validated test rules was launched on February 5, 2023, and the full validation process was completed by May 18, 2023, at which point auto-verification was fully implemented for all tests.

### TAT

The laboratory-defined TAT targets were 240 minutes for routine tests and 75 minutes for stat tests. TAT was measured as the time interval between sample acceptance and result verification within the laboratory. TAT values and TAT exceedance rates were calculated separately for the emergency department/intensive care units and the inpatient/outpatient clinics. These metrics were compared across equivalent one-month periods before and after the implementation of auto-verification. During these pre- and postimplementation periods, no major changes occurred in the number of analysers in operation, laboratory staff, or analytical platforms. The overall monthly test volume was comparable between the two periods.

### Statistical analysis

Data were analysed using the Statistical Package for the Social Sciences (SPSS), version 18.0 for Windows (SPSS Inc., Chicago, IL, USA). Auto-verification rates were calculated on both tube- and test-based bases. The frequency of rule violations leading to manual verification was evaluated using Pareto charts. The normality of data distribution was assessed using the Shapiro-Wilk test, and TAT was expressed as median (1^st^-3^rd^ quartile). The proportion of TAT exceedance before and after auto-verification was compared using the chi-square test. A p-value of <0.05 was considered statistically significant.

## Results

**Figure 2:**
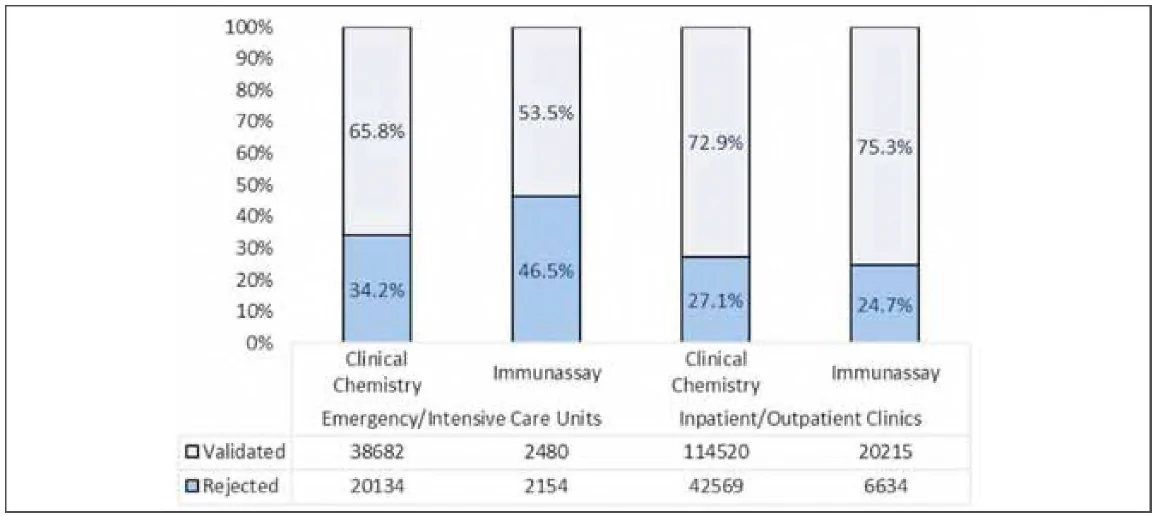
Test-based autoverification rates according to the clinics.

The test-based auto-verification rate was 71.1%, while the tube-based auto-verification rate was 21%. A total of 65% of emergency test results were verified automatically. The auto-verification rate in clinical chemistry tests was 65.8%, whereas it was 53.5% in immunoassay tests. In routine tests, the auto-verification rate was 73.3%, with 72.9% in clinical chemistry and 75.3% in immunoassay tests, as shown in Figure 2.

**Figure 3:**
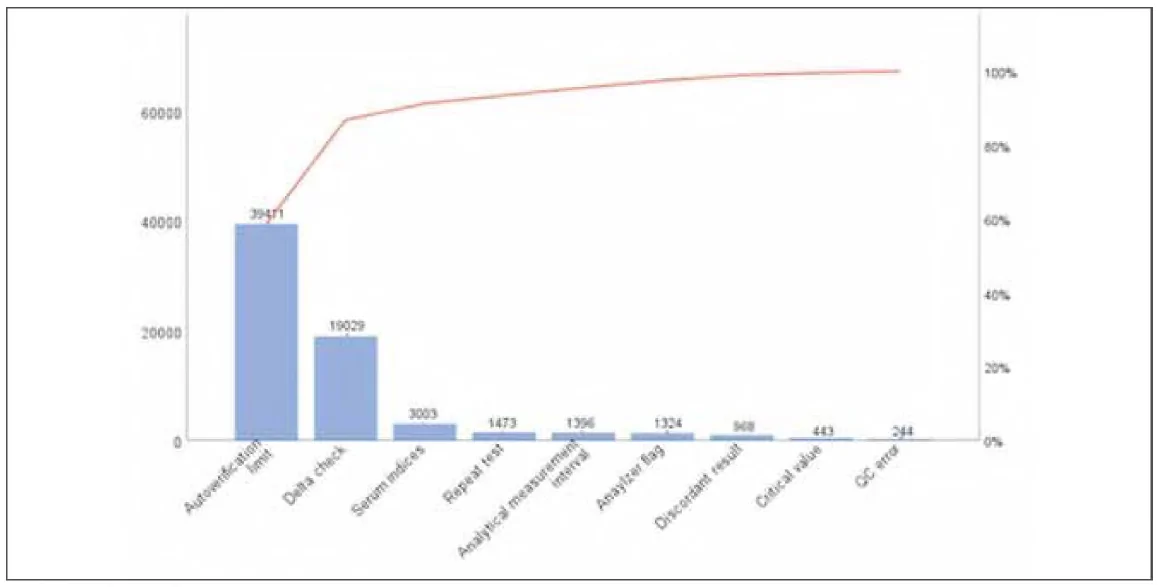
Rule violations in clinical chemistry tests.

**Figure 4:**
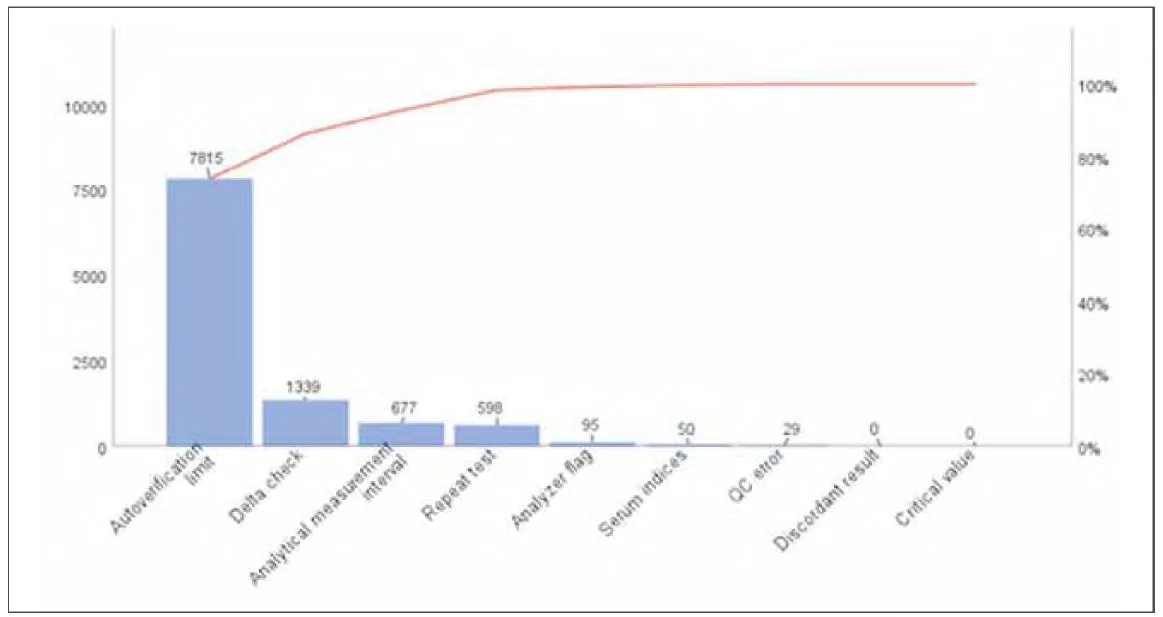
Rule violations in immunassay tests.

In clinical chemistry tests, the most common rule violations leading to manual verification were exceeding the auto-verification limit, followed by delta check failures and serum index interferences. In immunoassay tests, the most frequent causes were again exceeding the auto-verification limit, followed by delta check and analytical measurement interval violations, as illustrated in Figure 3 and Figure 4.

Among clinical chemistry parameters, LDL cholesterol had the highest auto-verification rate at 97.7%, followed by iron at 90.0% and sodium at 89.6%. The lowest auto-verification rates in this category were observed in glucose (47.9%), CRP (48.9%), and lactate (53.5%). In immunoassay tests, fT4 showed the highest auto-verification rate at 90.3%, followed by folate (89.6%) and vitamin B12 (89.4%). In contrast, pro-BNP had the lowest autoverification rate at 23.5%, followed by progesterone (38.1%) and ferritin (49%). Test-based validation approval rates for each analyte.

When the rate of TAT exceedance before and after auto-verification was compared, 6.4% of results in the pre-auto-verification period exceeded the target TAT, whereas this rate decreased to 5.8% in the post-auto-verification period, and this difference was statistically significant (p<0.001). Additionally, a 6-minute reduction in TAT was observed for emergency tests, and a 12-minute reduction for routine tests, as shown in Table 3.

**Table 3:** Turnaround times for pre and post-auto verification. TAT was presented as the median (1st-3rd quartile). p < 0.05 was considered statistically significant, obtained from the chi-square test. TAT, turnaround time; AV, auto verification.

Tests	Period	TAT (minutes)	The rate of exceeding TAT (%)	p-value
Emergency	Pre-AV	47 (37-65)	16.9%	<0.001
	Post-AV	41 (33-58)	14.2%	
Routine	Pre-AV	80 (53-124)	3.2%	<0.001
	Post-AV	68 (48-111)	2.9%	

## Discussion

This study presents our first experience implementing auto-verification for clinical chemistry and immunoassay tests. Given that modular systems were used in the laboratory, simultaneous processing of both test groups was targeted to achieve efficient and effective auto-verification.

Accurate and timely verification of results is one of the most critical responsibilities of clinical laboratories. However, in settings where the number of clinical biochemistry specialists and experienced technicians is limited, challenges such as delayed verification, inconsistencies between users, and potential errors in result interpretation may arise. These factors can lead to prolonged TAT and reduced reliability of laboratory reports. Furthermore, user-dependent variability in manual verification may compromise the standardisation of results. In a study by Gül et al. [18], agreement between seven independent reviewers and the auto-verification rules implemented for clinical chemistry tests ranged from minimal to moderate. Implementing standardised auto-verification protocols not only improves the consistency and objectivity of result evaluation but also enhances overall testing quality and ensures greater reliability in clinical decision-making.

Various studies have reported that auto-verification approval rates typically range from over 75% to, in rare cases, 95% [18][19][20]. These rates can vary depending on factors such as the rules defined within the system, the diversity of the test panels included, the characteristics of the patient population (e.g., emergency, inpatient, or outpatient), and national guideline requirements. In our laboratory, the test-based auto-verification rate was 71%, which is lower than rates reported in other studies. This difference may be attributed to our first implementation of autoverification and to the fact that we serve as a tertiary care centre, where a higher proportion of critically ill patients are tested.

Among clinical chemistry tests, the lowest auto-verification rates were observed in glucose, CRP and lactate. Although the glucose test was categorised into fasting plasma glucose and postprandial blood glucose in the automation system, the auto-verification rate remained low, mainly due to rule violations based on reference limits. This occurred because patients' fasting status was often unknown at the time of blood collection. In the case of CRP low auto-verification rates were largely due to frequent recurrences of abnormal values during serial monitoring in intensive care units and inpatient wards. Since plasma lactate level was typically required only in specific clinical conditions - such as metabolic acidosis or tissue hypoxia - the auto-verification rate was low. One major reason for the overall low auto-verification rate is that all rules operate concurrently; if any single rule is violated, auto-verification is halted. As a result, even repeat test results that are consistently pathological - but do not exceed delta check thresholds - are excluded from auto-verification. Creating and implementing specific rules to enable automated verification of repetitive, stable pathological results that fall outside standard limits could improve approval rates.

In the immunoassay group, proBNP progesterone, and ferritin had the lowest auto-verification rates. The low rate for proBNP was likely due to its targeted use in specific patient groups, while progesterone was primarily requested for patients undergoing hormone therapy at in vitro fertilisation centres, frequently resulting in values that exceeded defined verification thresholds.

One of the main reasons for the low tube-based auto-verification rate is that, in modular systems, both clinical chemistry and immunoassay tests are processed in a single tube and barcode, and are therefore reported together. As a result, both test groups are evaluated within the same report. Additionally, in a tertiary care setting with a broad test menu, frequent resident physician rotations, and new staff onboarding, unnecessary or excessive test ordering can occur. It has been reported that in a test panel containing 20 different parameters for a healthy individual, there is a 64% probability that at least one result will fall outside the reference range [21]. Although TEa was used to broaden the auto-verification limits in many tests, including multiple parameters in a single report, it indirectly lowers the tube-based auto-verification rate.

In addition to improving standardisation and reducing the manual verification workload, auto-verification also shortened laboratory TAT. The 6-minute reduction in TAT for emergency tests may help clinicians receive results earlier in conditions such as sepsis, acute glucose abnormalities, electrolyte imbalances, liver injury, and acute renal failure, where treatment decisions are time-sensitive. Likewise, the 12-minute reduction in routine TAT can support earlier decision-making during ward rounds and contribute to smoother outpatient workflows.

Given that the patient populations served by clinical laboratories vary across hospitals, each laboratory should design its own auto-verification algorithms tailored to its specific needs. Nonetheless, CLSI guidelines and minimum national regulatory frameworks can be utilised in constructing these algorithms [7][12]. In our case, algorithm development was based on such recommendations. Auto-verification is a dynamic process and requires continuous monitoring and refinement using real patient data. During the validation phase, the presence of a comment column next to each result in the LIS facilitated simultaneous manual verification and observation of the auto-verification system's performance. This setup enabled immediate intervention for malfunctioning rules. For example, during the initial rule development, the auto-verification limits for electrolytes were defined by expanding the reference intervals using TEa. However, during validation, it was observed that minor fluctuations - such as voltage instability or changes in water conductivity - could influence the analytical process and slightly alter results without exceeding delta check limits. While the number of affected samples was small, auto-verification continued to approve these results, and the issue was identified during manual review. In response, the auto-verification criteria were revised to define the verification limits strictly based on the reference intervals rather than TEa. For certain parameters, such as ammonia, CRP total and direct bilirubin, RF, and ASO values outside the analytical measurement range were initially flagged as rule violations. However, when results below the lower analytical measurement limit were consistent with previous values, they were verified manually without issue. Consequently, the algorithm was revised so that only values exceeding the upper analytical measurement limit were considered rule violations for auto-verification in these tests. A similar approach was reported by Miler et al., who validated their system by viewing how auto-verification performed in real time through a dedicated column in the LIS [19].

During the installation phase of the auto-verification system, access to the middleware program from the LIS was limited due to data security concerns. As a result, patient clinical information could not be transferred to the middleware, and diagnosis-specific algorithms could not be developed. For instance, results below the analytical measurement range in patients undergoing prostatectomy, or low fT4 levels alongside elevated TSH in hypothyroid patients, required manual validation, as no customised auto-verification rules could be created for these clinical scenarios. Another limitation was the initial lack of inpatient/outpatient status information within the middleware. This omission prevented the application of department-specific critical value thresholds - for example, the absence of a defined panic value for creatinine in patients receiving haemodialysis - thereby limiting the full potential of auto-verification in such cases.

A further shortcoming of our algorithm was the lack of real-time, patient-based quality control. Establishing dynamic quality control rules based on patient results, particularly for calcium, magnesium, and electrolytes, in outpatient clinics could significantly improve the analytical phase and enable more precise result verification.

Although the auto-verification algorithms were prospectively validated in the live system on a test-bytest basis through real-time observation and correction of rule violations, a parallel comparison of manual and auto-verification decisions was not performed for all evaluated samples. Therefore, false acceptance and false rejection rates could not be calculated, which represents an important limitation of this study.

In this study, we presented the auto-verification rules, the algorithm, and the implementation experience in a university hospital setting. Despite being an initial implementation and facing certain limitations, auto-verification enabled more time and attention to be dedicated to manual verification of critical test results and significantly improved the post-analytical phase. Moreover, it contributed to preanalytical improvements through rules such as EDTA contamination detection and enhanced the analytical phase via analyser-generated flags, making the total testing process more effective and reliable. Expanding auto-verification to include additional test groups, such as haematology, coagulation, and urinalysis, will further increase patient-based auto-verification report rates. This, in turn, will allow clinical biochemistry specialists to allocate more time to rational test utilisation practices, clinical consultations, and the training of laboratory staff.

## Dodatak

### List of abbreviations

ALP alkaline phosphatase;<br>ALT, alanine aminotransferase;<br>ASO anti-streptolysin O;<br>AST, aspartate aminotransferase;<br>CLIA, clinical laboratory improvement amendments;<br>CLSI, clinical and laboratory standards institute;<br>CRP, C-reactive protein;<br>CVA, analytical variation;<br>CVW, within-subject biological variation;<br>EFLM, European Federation of Clinical Chemistry and Laboratory Medicine;<br>fT3, free tri-iodothyronine;<br>fT4, free thyroxine;<br>FSH, follicle-stimulating hormone;<br>GGT, gamma-glutamyltransferase;<br>HDL, high-density lipoprotein;<br>LDH, lactate dehydrogenase;<br>LDL, low-density lipoprotein;<br>LIS, laboratory information system;<br>LH, luteinising hormone;<br>pro-BNP, pro-brain natriuretic peptide;<br>PSA, prostate-specific antigen;<br>PTH, parathyroid hormone;<br>QC, quality control;<br>RCV, reference change value;<br>RF, rheumatoid factor;<br>SPSS, statistical package for the social sciences;<br>TAT, turnaround time; tea, total allowable error;<br>TSH, thyroid-stimulating hormone.

### Acknowledgements

I want to express my sincere gratitude to Mehmet Helvaci, Ozan Ciner, and the Roche Diagnostics IT & Digital Solutions Squad for their valuable support in implementing the algorithms and establishing the connection with the LIS. I also extend my thanks to Sevgi §im§ek and Ozay Tuncay for their assistance and dedication during the validation phase.

### Conflict of interest statement

All the authors declare that they have no conflict of interest in this work.
